# Species-Specific Heterochromatin Prevents Mitotic Chromosome Segregation to Cause Hybrid Lethality in *Drosophila*

**DOI:** 10.1371/journal.pbio.1000234

**Published:** 2009-10-27

**Authors:** Patrick M. Ferree, Daniel A. Barbash

**Affiliations:** Department of Molecular Biology and Genetics, Cornell University, Ithaca, New York, United States of America; Duke University, United States of America

## Abstract

Postzygotic reproductive barriers such as sterility and lethality of hybrids are important for establishing and maintaining reproductive isolation between species. Identifying the causal loci and discerning how they interfere with the development of hybrids is essential for understanding how hybrid incompatibilities (HIs) evolve, but little is known about the mechanisms of how HI genes cause hybrid dysfunctions. A previously discovered *Drosophila melanogaster* locus called *Zhr* causes lethality in F1 daughters from crosses between *Drosophila simulans* females and *D. melanogaster* males. *Zhr* maps to a heterochromatic region of the *D. melanogaster* X that contains 359-bp satellite repeats, suggesting either that *Zhr* is a rare protein-coding gene embedded within heterochromatin, or is a locus consisting of the noncoding repetitive DNA that forms heterochromatin. The latter possibility raises the question of how heterochromatic DNA can induce lethality in hybrids. Here we show that hybrid females die because of widespread mitotic defects induced by lagging chromatin at the time during early embryogenesis when heterochromatin is first established. The lagging chromatin is confined solely to the paternally inherited *D. melanogaster* X chromatids, and consists predominantly of DNA from the 359-bp satellite block. We further found that a rearranged X chromosome carrying a deletion of the entire 359-bp satellite block segregated normally, while a translocation of the 359-bp satellite block to the Y chromosome resulted in defective Y segregation in males, strongly suggesting that the 359-bp satellite block specifically and directly inhibits chromatid separation. In hybrids produced from wild-type parents, the 359-bp satellite block was highly stretched and abnormally enriched with Topoisomerase II throughout mitosis. The 359-bp satellite block is not present in *D. simulans*, suggesting that lethality is caused by the absence or divergence of factors in the *D. simulans* maternal cytoplasm that are required for heterochromatin formation of this species-specific satellite block. These findings demonstrate how divergence of noncoding repetitive sequences between species can directly cause reproductive isolation by altering chromosome segregation.

## Abstract 2

Early embryonic lethality of interspecies hybrids in *Drosophila* can be caused by defects in mitotic segregation of paternal X chromatids carrying a critical domain of heterochromatic DNA.

## Author Summary

Speciation is most commonly understood to occur when two species can no longer reproduce with each other, and sterility and lethality of hybrids formed between different species are widely observed causes of such reproductive isolation. Several protein-coding genes have been previously discovered to cause hybrid sterility and lethality. We show here that first generation hybrid females in *Drosophila* die during early embryogenesis because of a failure in mitosis. However, we have discovered that this is not a general failure in mitosis, because only the paternally inherited X chromosome fails to segregate properly. Our analyses further demonstrate that this mitotic failure is caused by a large heterochromatic region of DNA (millions of base pairs) that contains many repetitive copies of short noncoding sequences that are normally transcriptionally quiescent. Interestingly, this block of heterochromatin is only found in the paternal species. We suggest that a failure of the maternal species to package this paternally inherited DNA region into heterochromatin leads to mitotic failure and hybrid lethality. If this is a general phenomenon it may explain other examples of hybrid lethality in which F1 females die but F1 males survive.

## Introduction

A critical stage of speciation is the development of reproductive isolating mechanisms that prevent gene exchange between diverging populations. Hybrid sterility and lethality are major components of reproductive isolation. A key to understanding how these hybrid incompatibilities (HIs) evolve is discovering the causal genes and determining how they inhibit or perturb normal development. A number of HI genes have been identified, all of which are protein-coding. These genes are characterized by two distinct modes of evolution: either high rates of coding-sequence divergence that are consistent with adaptive evolution in many [1]–[3] but not all [4] cases, or structural changes such as in gene location [5] or gene silencing and loss following duplication [6],[7]. These cases suggest that rapid evolution of either protein-coding gene sequence or structure is a general principle underlying the evolution of HIs.

Are rapidly evolving protein-coding genes the only cause of HI? Noncoding repetitive sequences, including transposable elements (TEs) and satellite repeats, are major contributors to genome evolution in higher eukaryotes. These sequences comprise heterochromatin, chromosomal regions found primarily around the centromeres and telomeres that remain more condensed than gene-containing euchromatin through the cell cycle. Pericentric heterochromatin is known to play important roles in mitotic and meiotic chromosome segregation [8]–[10]. Heterochromatin may also be important for the transcriptional regulation of flanking sequences such as ribosomal DNA (rDNA) loci, since rDNA genes are often found in heterochromatic regions [11],[12]. Paradoxically, however, despite these apparently conserved functions in higher eukaryotes, heterochromatin can vary greatly in abundance and sequence composition even between closely related species [13]–[16]. These observations have led to speculation that divergence of repetitive noncoding sequences may also directly cause reproductive isolation between nascent species [13],[17]. However, to our knowledge no examples have been clearly demonstrated.

One hint that heterochromatin divergence may contribute to HI came from the discovery that the protein encoded by the *Drosophila* hybrid lethality gene *Lhr* localizes to pericentric heterochromatin. *Lhr* itself shows strong evidence of having diverged under the force of adaptive evolution, leading to the hypothesis that it may be co-evolving with heterochromatic sequences [18]. An additional possible link between HI and heterochromatin comes from the identification of the gene *Prdm9* as causing hybrid male sterility between subspecies of mice, because the heterochromatic meiotic sex body is defective in both sterile hybrids and in *Prdm9*-mutant pure-species mice [19].

The sibling species *D. melanogaster* and *D. simulans* exhibit large differences in heterochromatin content [15] and strong reproductive isolation [20]. F1 hybrid females produced from *D. simulans* mothers and *D. melanogaster* fathers die as embryos [21]. This female-specific lethality is intriguing for several reasons. First, this lethality appears to have a different genetic basis than the F1 male lethality that occurs in the reciprocal cross [22]. While two major-effect genes causing this male lethality have been cloned [18],[23], nothing is known about the molecular basis of the female lethality. Second, this female-specific lethality is an exception to Haldane's rule, the observation that unisexual hybrid sterility or lethality typically affects the heterogametic (XY or ZW) sex rather than the homogametic sex (XX or ZZ) [24].

Third, a link between hybrid female lethality and heterochromatin was strongly suggested by studies of Sawamura and colleagues of the *D. melanogaster Zygotic hybrid rescue* (*Zhr* ^1^) mutation, which suppresses lethality of these otherwise lethal hybrid females. *Zhr* ^1^ was discovered on an X-Y translocation chromosome that is deleted for much of the X chromosome pericentric heterochromatin [25]. The deleted region is thought to consist primarily of satellite DNA composed of a tandemly repeated 359-bp long monomer [25]. We refer henceforth to the monomer unit as the 359-bp repeat, and the heterochromatic region of the *D. melanogaster* X chromosome as the 359-bp satellite block, and revisit in the Discussion the question of what specific DNA sequences within this block cause hybrid lethality. In the wild type this satellite DNA (also known as the 1.688 g/cm^3^ satellite) is estimated to form a multi-mega-bp block of heterochromatin [26]. Experiments showing that hybrid viability is sensitive to the dosage of a mini-chromosome containing part of the 359-bp satellite block led to the suggestion that repetitive sequences within the 359-bp satellite block are responsible for the hybrid lethal effect [27]. However, the mapping studies are consistent with the alternative possibility that the *Zhr* locus is a protein-coding gene embedded within this heterochromatic region. This is a plausible alternative, as an unexpected number of protein-coding genes have recently been found on *Drosophila* Y chromosomes, which otherwise contain mega-bp amounts of heterochromatic repeats [28].

If *Zhr* is not a protein-coding locus, then the possibility that an HI locus consists of noncoding, repetitive DNA raises important questions regarding how such sequences could kill hybrids. One possibility is that heterochromatic sequences such as those comprising the X-linked *Zhr* locus cause hybrid lethality by inducing in *trans* a global effect on chromatin structure or gene expression. Alternatively, the *Zhr* locus might operate in *cis* by affecting other adjacent, X-linked sequences such as the rDNA genes or the centromere. A third alternative is that the lethal effects are confined to this heterochromatic locus itself, such that an aberration in its structure somehow directly disrupts embryonic development.

Given the difficulties involved in the genetic manipulation of heterochromatic sequences, we addressed these questions by combining genetic and cytological approaches to determine when hybrid females die during development, to identify the cellular basis of the lethality, to investigate whether possible heterochromatic defects occur genome-wide or are confined to the *Zhr* locus, and to test whether such defects are suppressed in hybrid females carrying the *Zhr* ^1^ rescue mutation and induced in hybrid males carrying a *Zhr* duplication. Our results strongly suggest that the *Zhr* locus directly causes hybrid lethality by inducing mitotic failure in early precellularized embryos, and that the underlying defect is a failure of the 359-bp satellite block to form or maintain a proper heterochromatic state. These results provide compelling evidence that noncoding heterochromatic DNA can directly cause HI and thus contribute to speciation.

## Results

### Hybrid Females Exhibit Mitotic Asynchrony and Lagging Chromatin during a Discrete Period of Early Embryogenesis

**Figure 1:**
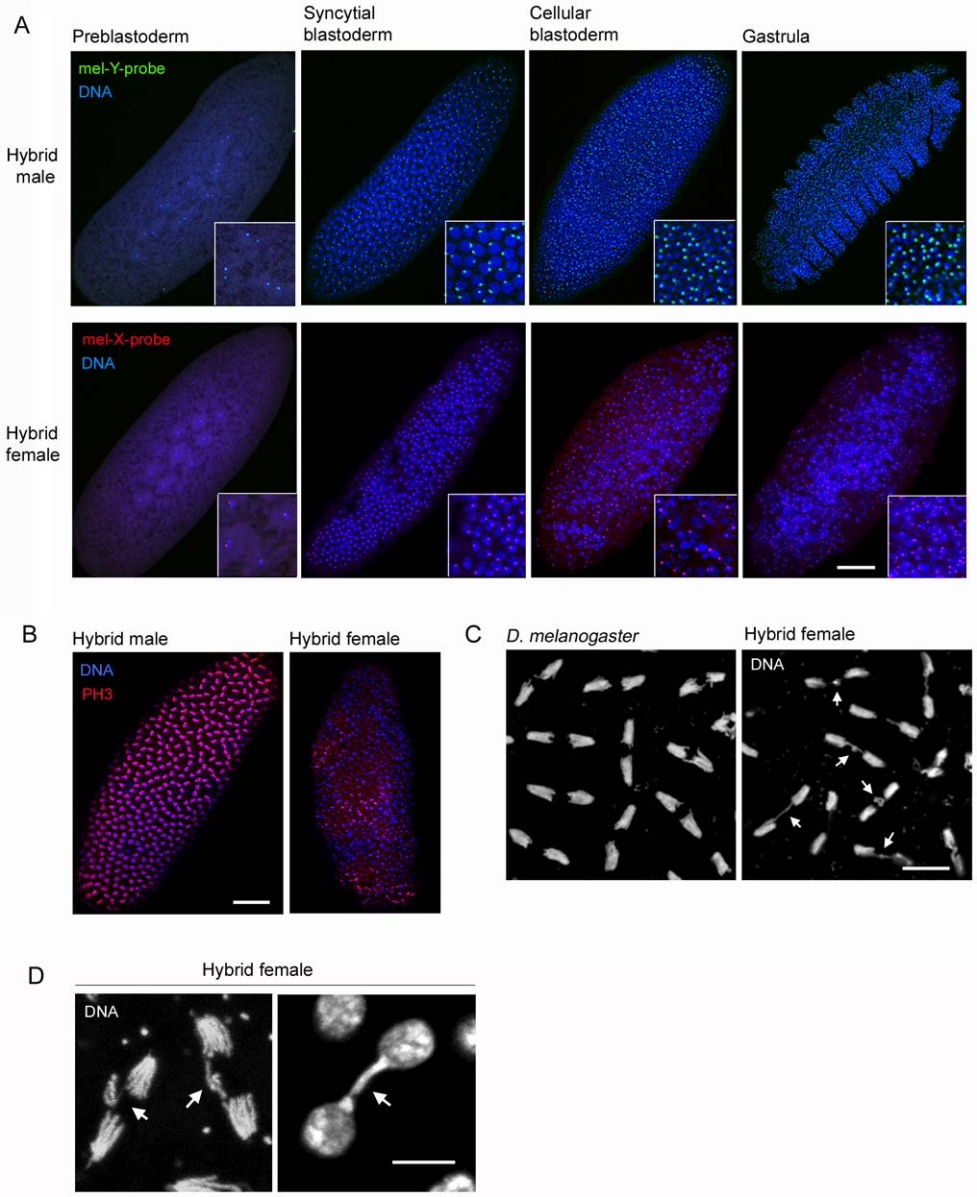
Young hybrid female embryos exhibit defects in nuclear spacing, mitotic asynchrony, and lagging chromatin. (A) Hybrid male and female embryos at different stages of early embryogenesis. Embryos were sexed using FISH probes to the *D. melanogaster* X (359-bp satellite) and Y (AATAGAC satellite) chromosomes. Higher magnifications of the nuclei are shown in the insets. Hybrid male embryos develop normally, while hybrid female embryos show abnormal nuclear spacing beginning during nuclear divisions 10–13 of the syncytial blastoderm stage. (B) Hybrid female embryos exhibit mitotic asynchrony. Left panel, a hybrid male embryo showing wild-type pattern. Right panel, a hybrid female embryo. Both embryos were stained with anti-phospho-Histone 3 (PH3) antibodies. (C) Chromosome mis-segregation in hybrid female embryos. White arrows in right panel indicate lagging chromatin during anaphase in a hybrid female embryo. (D) Left panel is a high magnification of two dividing chromosome sets during anaphase. Right panel is a high magnification of two daughter nuclei during late telophase. White arrows indicate lagging chromatin. Scale bar is 90 µm in (A) and (B), 8 µm in (C), and 5 µm in (D).

To address the timing and nature of hybrid female lethality, we examined young (0–3 h) hybrid embryos produced from several different wild-type parental strains (Table 1). Normal embryonic development in *Drosophila* begins with a single diploid nucleus that gives rise to several thousand nuclei through 14 synchronous mitotic divisions in the large, single-celled blastula. During the first nine divisions, the nuclei migrate from the interior of the embryo to the cortex as they expand in number. Four additional nuclear divisions occur at the cortex before the formation of membrane furrows that transform the syncytial blastoderm into the cellular blastoderm. This process, termed cellularization, is followed by gastrulation (for a detailed review of early *Drosophila* embryogenesis see [29]). As expected, hybrid male embryos, which survive to adulthood [20], underwent normal nuclear divisions during the blastula stage and progressed into the gastrula stage (Figure 1A). Hybrid female embryos also had normal nuclear spacing and synchrony during the first nine mitotic divisions (Figure 1A). However, between mitotic divisions 10–13, hybrid female embryos exhibited large areas near the cortex devoid of nuclei and abnormal amounts of nuclei remained deep within the cytoplasm, indicating a high level of failed nuclear divisions (Figure 1A). The nuclei at the cortex were irregularly shaped and spaced (Figure 1A) and stained unevenly for the mitotic marker phospho-Histone-3 (PH3) (Figure 1B), demonstrating that these nuclei have asynchronous cell cycles.

**Table 1:** Hybrid female viability from crosses between different *D. simulans* and *D. melanogaster* strains.

Cross (*D. simulans* female×*D. melanogaster* male)	*n* Hybrid Progeny		Percent Hybrid Females
	Males	Females	
*white^501^ (SA32)*×Canton S	232	13	5.3
C167.4×Canton S	525	39	6.9
C167.4 (tet)×Canton S^a^	233	34	12.7
C167.4×Oregon R	310	43	12.2
NC48S×Canton S	180	0	0
NC48S×*Zhr* ^1^	325	392	54.7
NC48S×*Zhr* ^1^/*Zhr* ^+^ Y	48	112	70.0

a The C167.4 line was found to be infected with *Wolbachia* bacteria. This line was treated with antibiotics to produce the C167.4 (tet) line, which is cured of *Wolbachia*. Interspecific crosses with this line showed that *Wolbachia* do not influence hybrid female lethality.

We also observed lagging chromatin between the dividing chromosome sets during anaphase and telophase in hybrid female embryos (Figure 1C and 1D). Lagging chromatin was observed in all analyzed hybrid female embryos (*n* = 16), ranging from 40% (13/32) to 100% (11/11) aberrant anaphase spindles per embryo, which is consistent with the high hybrid female lethality (∼87%–100%) produced from these crosses (Table 1). It is likely that the lagging chromatin is the direct cause of the mitotic asynchrony and other nuclear defects in hybrid female embryos, an idea supported by studies showing that mutations causing chromosome bridges lead to similar mitotic defects in *D. melanogaster* embryos [30]–[32].

### The *D. melanogaster* X Chromatids Fail to Separate during Anaphase in Hybrid Female Embryos

**Figure 2:**
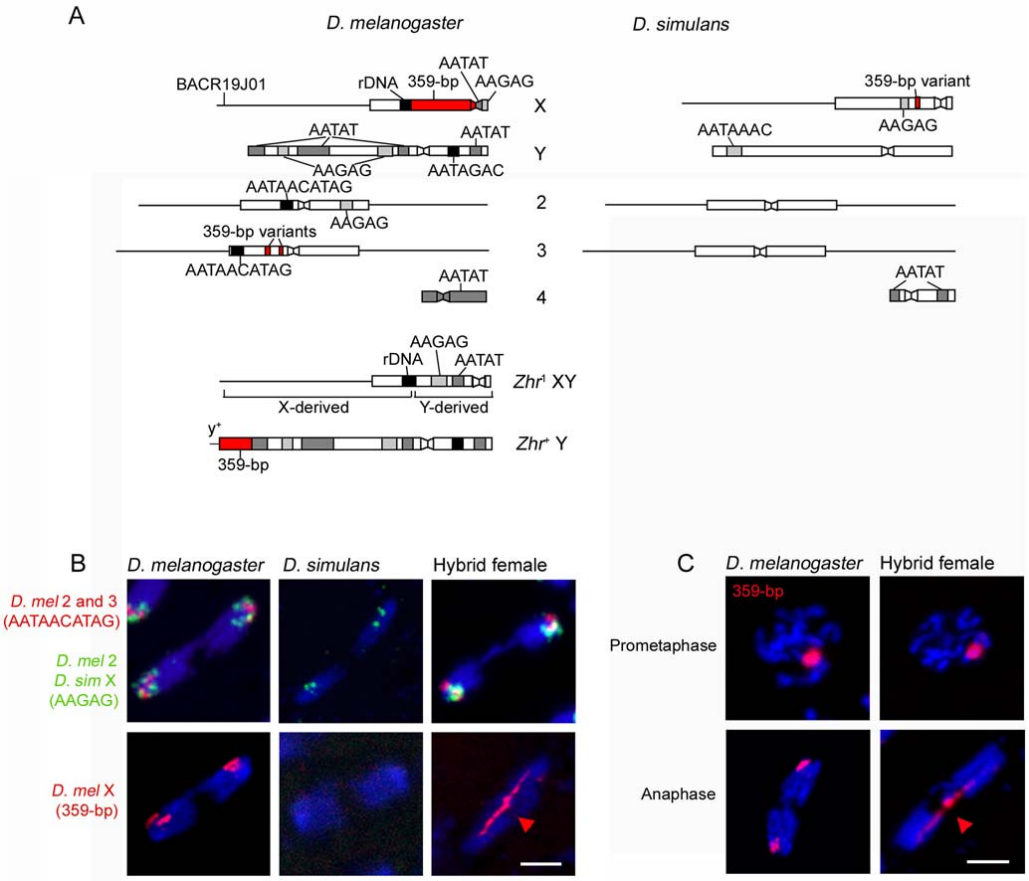
Separation failure of the *D. melanogaster* X chromatids during anaphase in hybrid female embryos. (A) Schematic of satellites and other loci used as targets of FISH, and of *Zhr* mutant and duplicated chromosomes. Mapping of FISH probes is shown in Figure S2. Boxes and lines represent heterochromatin and euchromatin, respectively. Constrictions in boxes represent the centromeres. Chromosomal regions are not drawn to scale. The *Zhr* ^1^ chromosome is thought to have resulted from a recombination between an X and a Y [25]. Our FISH analysis shows that this compound-XY chromosome contains the Y centromere and the proximal half of the Y long arm fused with the X euchromatin and distal pericentric heterochromatin (Figure S2); note that this structure differs from that inferred by Sawamura et al. [25]. In this chromosome, the entire 359-bp satellite block has been deleted. The *Zhr* ^+^ Y chromosome was made by a translocation onto the distal end of the Y long arm of half of the X-derived 359-bp satellite block and a small amount of distal euchromatin carrying the *y* ^+^ marker from an inverted X chromosome (*In(1)sc* ^8^) [36]. (B) Top row, *D. melanogaster* 2 and 3 chromatids and the *D. simulans* X chromatids segregate normally in hybrid female embryos. Bottom row, the *D. melanogaster* X chromatids, marked by a probe for the 359-bp satellite (red arrowhead), fail to segregate and comprise the lagging chromatin. (C) The 359-bp satellite block is normally condensed during prometaphase but abnormally stretched (red arrowhead) during anaphase in hybrid female embryos. DNA is blue in (B) and (C). Scale bar is 5 µm in (B) and (C).

To determine whether the lagging chromatin in hybrid female embryos results from a general defect in chromosome segregation or is instead chromosome-specific, we performed fluorescent in situ hybridization (FISH) with probes that recognize distinct satellite sequences in the pericentric regions of different *D. melanogaster* and *D. simulans* chromosomes (Figure 2A). Probe signals for sequences on *D. melanogaster* Chromosomes 2 and 3 and the *D. simulans* X chromosome were found in condensed regions near the spindle poles and never within the lagging chromatin (*n* = 75/75 spindles from 13 embryos; Figure 2B), indicating normal segregation of these chromosomes.

We also analyzed the segregation of the *D. melanogaster* X chromosome in hybrid female embryos by using a probe for the 359-bp repeat. The 359-bp repeat probe labeled two abnormally stretched strands leading outward from the lagging chromatin toward opposite spindle poles (*n* = 56/100 spindles from nine embryos; Figure 2B). Stretched 359-bp repeat DNA was also observed in anaphase spindles in which there was no lagging chromatin (*n* = 37/100 spindles; Figure S1). Our mapping of the 359-bp repeat probe on chromosome spreads from larval brain tissue confirmed the presence of the major block of 359-bp satellite located on the *D. melanogaster* X, as well as several minor blocks of related satellites (353-bp, 356-bp, and 361-bp repeats) on the left arm of *D. melanogaster* Chromosome 3 (also see Figure S2) [33]. These smaller regions appeared unstretched and segregated normally in hybrid female embryos (Figure S3). A variant of the 359-bp repeat is also present in a small satellite block in the pericentric region of the *D. simulans* X chromosome (Figure S2) but does not cross-hybridize with the 359-bp repeat probe under our experimental conditions (Figures 2B and S2), presumably because of its high level of sequence divergence from the *D. melanogaster* repeats [34]. The lagging chromatin in hybrid female embryos, therefore, is derived solely from the *D. melanogaster* X chromosome. Moreover, this stretching effect likely results from partial or complete failure of the sister *D. melanogaster* X chromatids to separate during anaphase rather than from defective X chromatin condensation because the 359-bp satellite block appeared properly condensed during metaphase (Figure 2C).

### The 359-Bp Satellite Block Prevents *D. melanogaster* X Chromatid Separation in Hybrids

**Figure 3:**
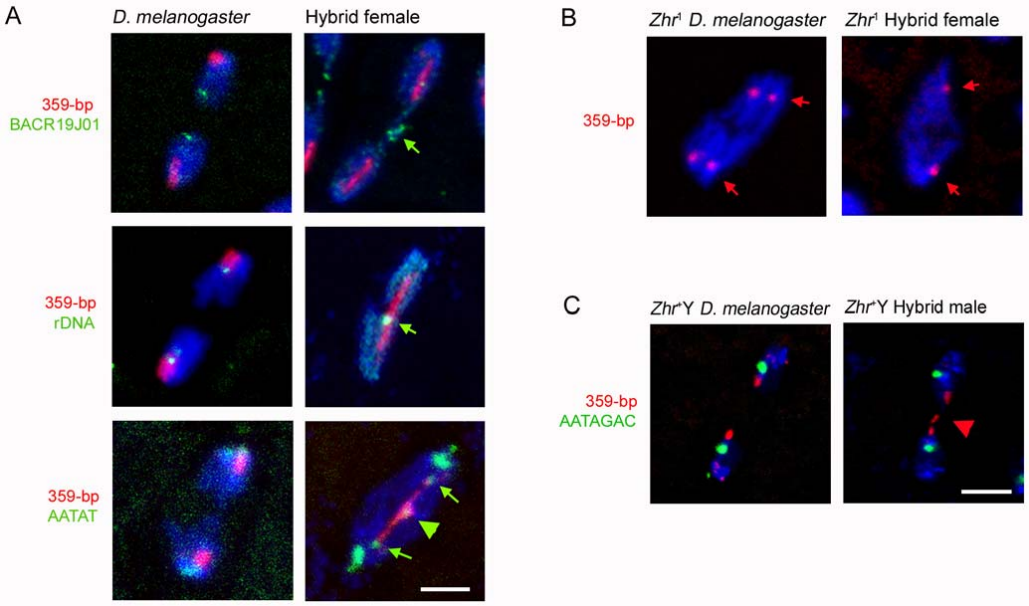
Stretched and lagging X heterochromatin is induced by sequences in the 359-bp satellite block in hybrid female embryos. (A) Green arrows in right top and middle panels indicate unstretched euchromatin and rDNA, respectively. In the right bottom panel, green arrows highlight AATAT satellite DNA proximal to the 359-bp DNA block that is segregating to the spindle poles, while the green arrowhead indicates a small amount of stretched and lagging AATAT DNA. (B) Normal X chromosome segregation in hybrid females carrying the *Zhr* ^1^ compound-XY chromosome, which is devoid of the X-linked 359-bp satellite. The related satellites (353-bp, 356-bp, and 361-bp repeats, indicated by red arrows) on Chromosome 3 segregate normally. (C) 359-bp DNA translocated to the Y chromosome is stretched and lagging in a hybrid male embryo (right panel). The *D. melanogaster* control embryo carries the *Zhr* ^1^ compound-XY chromosome instead of a wild-type X chromosome, so that the only source of 359-bp satellite is the Y. The red arrowhead points to lagging Y-linked 359-bp satellite DNA. DNA is blue in all panels. Scale bar is 5 µm in (A) and 7 µm in (C).

We used FISH with additional probes to determine whether separation failure of the *D. melanogaster* X chromatids is confined to the 359-bp satellite block or occurs in other regions of this chromosome. Probe signals from a euchromatic region located at the distal end (cytogenetic location 1C3-4) of the major left arm and from the tandemly repeated rDNA genes (*bobbed* ^+^ locus) in the distal pericentric heterochromatin appeared as unstretched and condensed foci (for euchromatic region, *n* = 28/28 spindles from six embryos; for rDNA locus, *n* = 13/13 spindles from eight embryos; Figure 3A). A discrete signal of the simple-repeat satellite AATAT, which spans a portion of the minor right arm and part of the centromere immediately adjacent to the large 359-bp satellite block [35], was present at each end of the stretched 359-bp satellite block near the spindle poles in a pattern similar to the centromeric regions of the other chromosomes (Figure 3A). Therefore, the centromeres of the sister *D. melanogaster* X chromatids are active and separate at anaphase. However, we also observed small amounts of AATAT DNA stretched across the spindle and in the lagging chromatin, similar to the 359-bp satellite block (*n* = 20/34 spindles from three embryos; Figures 3A and S4). These results demonstrate that the stretched DNA is confined to the proximal X pericentric heterochromatin containing 359-bp and AATAT satellites, suggesting that sequences in this region are responsible for separation failure of the *D. melanogaster* X chromatids.

To determine the particular causal region of the pericentric heterochromatin, we examined the segregation of the *Zhr* ^1^ compound-XY chromosome (Figure 2A) in hybrid female embryos. Consistent with previous results [25], crosses between wild-type *D. simulans* females and *D. melanogaster Zhr* ^1^ males resulted in full viability of F1 hybrid female adults (Table 1). Our analysis of larval brain chromosome spreads from the *Zhr* ^1^ strain revealed that the compound-XY chromosome is completely devoid of the 359-bp satellite block but contains Y-derived AATAT repeats (Figure S2). In hybrid female embryos the *Zhr* ^1^ compound-XY chromosome segregated normally, as indicated by the complete absence of lagging chromatin during anaphase (*n* = 68/68 spindles from six embryos; Figure 3B). Furthermore, these embryos advanced properly through subsequent developmental stages into adulthood.

We also analyzed hybrid male embryos whose Y chromosome carries a translocation of approximately half of the X-linked 359-bp satellite block to the Y long arm (see Figure 2A) [36]. This *Zhr^+^* chromosome resulted in hybrid male lethality that was less severe than hybrid female lethality induced by the wild-type X chromosome (Table 1). A subset of these hybrid male embryos (4/14) exhibited mitotic asynchrony and lagging chromatin during anaphase and telophase (*n* = 34/45 spindles; Figure 3C), similar to but not as common as the defects described above in hybrid females. Moreover, FISH analysis showed that the chromatin bridges were comprised of Y-derived 359-bp repeat DNA in these hybrid males (Figure 3C). These results, together with our analyses of the *Zhr* ^1^ chromosome, strongly suggest that sequences contained specifically within the 359-bp satellite block induce chromosomal segregation failure in hybrid embryos.

### D1 Protein Does Not Localize to the 359-Bp Satellite Block in Embryos

Segregation failure of the *D. melanogaster* X chromatids in hybrid females occurs between nuclear cycles 10–13, a period when embryonic development is primarily under control of maternally contributed RNA and proteins [37]. Our findings, therefore, suggest that the *D. simulans* maternal cytoplasm lacks factors that are compatible with and necessary for proper segregation of the *D. melanogaster* X-linked 359-bp satellite block. This hypothesis is consistent with the fact that hybrid females produced from the reciprocal cross, carrying the 359-bp satellite block and the *D. melanogaster* maternal cytotype, are fully viable [20]. We therefore investigated the localization patterns of D1 and Topoisomerase II (TopoII), two proteins known to associate with the 359-bp satellite block in *D. melanogaster* [38]–[40].

**Figure 4:**
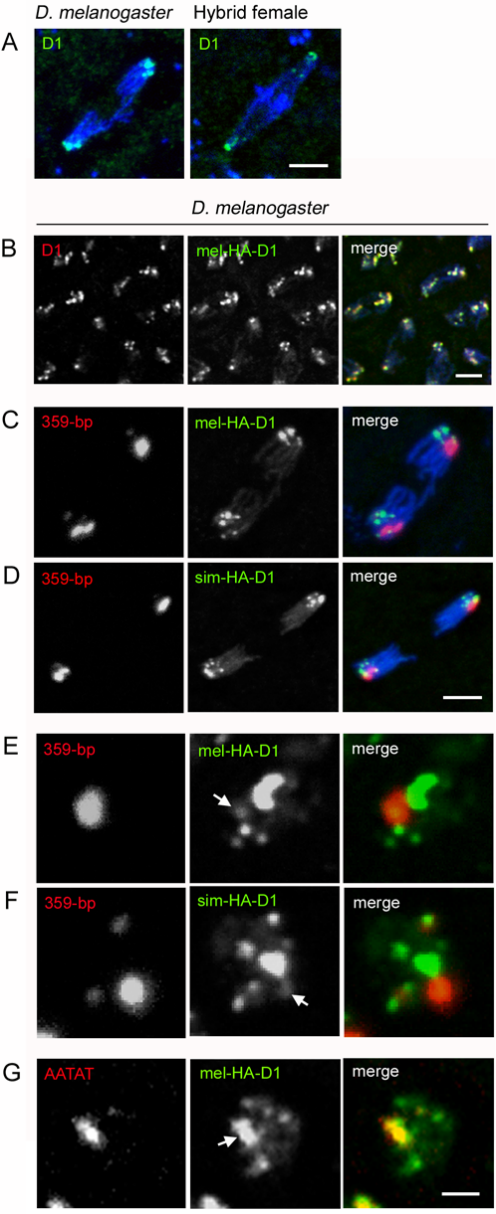
D1 localizes to AATAT satellite DNA but not the 359-bp satellite block during early embryogenesis. (A) Left panel, an anaphase spindle in a *D. melanogaster* embryo. Anti-D1 highlights endogenous D1, which localizes to pericentromeric regions near the spindle poles. Right panel, an anaphase spindle in a hybrid female embryo. Endogenous D1 exhibits a pattern similar to that found in *D. melanogaster*. DNA is blue in both panels. (B) Anaphase spindles from a *D. melanogaster* embryo with HA-tagged *D. melanogaster* D1 (*UAS-mel-HA-D1* driven by *P{matα4-GAL-VP16}V37*). Anti-D1 recognizes both endogenous and HA-D1. The two signals overlap completely, showing that the HA-D1 protein localizes identically to endogenous D1. (C) Anaphase spindle from the same genotype in (B), showing mel-HA-D1 and 359-bp DNA. (D) Anaphase spindle from a *D. melanogaster* embryo with HA-tagged *D. simulans* D1 (*UAS-sim-HA-D1* driven by *P{matα4-GAL-VP16}V37*). Neither of the D1 orthologs, (C) and (D), co-localizes with the 359-bp satellite block. (E) Interphase nucleus from the same genotype in (C). (F) Interphase nucleus from the same genotype in (D). Both D1 orthologs show only a slight co-localization with the 359-bp satellite block during interphase. White arrows in (E) and (F) indicate this minimal co-localization. (G) Interphase nucleus from the same genotype in (C). The mel-HA-D1 protein co-localizes strongly with AATAT satellite DNA (indicated by white arrow). Scale bar is 4 µm in (A), 5 µm in (B), 4 µm in (C, D) and 3 µm in (E–G).

Previous studies showed that the protein D1 localizes to AT-rich heterochromatin, including the 359-bp and AATAT satellites, in larval mitotic tissues [39],[40]. Additionally, D1 was found to influence the localization of heterochromatin protein 1 (HP1) to the 359-bp satellite block [40]. On the basis of these results, it was suggested that D1 may be a structural heterochromatin component of these satellites. To determine if D1 plays a role in the defective structure of the 359-bp satellite block in hybrids, we analyzed the localization of D1 in wild-type *D. melanogaster* and hybrid embryos with an antibody raised against *D. melanogaster* D1 [39]. In Western blots, this antibody recognized a single band of approximately 60 kDa, the predicted size of D1 in both *D. melanogaster* and *D. simulans* (Figure S5). In *D. melanogaster* and hybrid embryos, D1 was present during anaphase at numerous sites near the spindle poles, which are likely the AT-rich satellites in the centric and pericentric regions (Figure 4A). However, in hybrid female embryos, we observed no D1 localized to the lagging chromatin containing the 359-bp DNA (Figure 4A). These observations suggested the possibility that *D. simulans* D1 fails to bind these sequences in hybrids.

To test this hypothesis, we expressed *D. melanogaster* and *D. simulans* D1 in *D. melanogaster* embryos using the GAL4-UAS system (see Materials and Methods). Transgenic D1 localized to pericentric regions that completely overlapped with endogenous D1 (Figure 4B). We performed immuno-FISH experiments to simultaneously visualize *D. melanogaster* or *D. simulans* D1 with several satellite sequences. Both orthologs exhibited identical binding patterns in young embryos (Figure 4C–4F). Contrary to the prominent localization of D1 to 359-bp DNA in larval mitotic cells (also see Figure S2) [40], we observed barely detectable levels of D1 on this satellite block (Figure 4C–4F). Instead, D1 localized primarily to AATAT satellite DNA (Figure 4G). We propose that the major foci of D1 detected in embryos in earlier studies [39] and presumed to correspond with the 359-bp satellite block actually represent the large regions of AATAT on Chromosome 4. Our results demonstrate that unlike in larval brain cells, D1 is not a major component of the 359-bp satellite block during early embryogenesis, and likely does not play a role in the 359-bp structural defects observed in hybrid female embryos.

### Topoisomerase II Remains Abnormally Enriched on the 359-Bp Satellite Block in Hybrid Females

**Figure 5:**
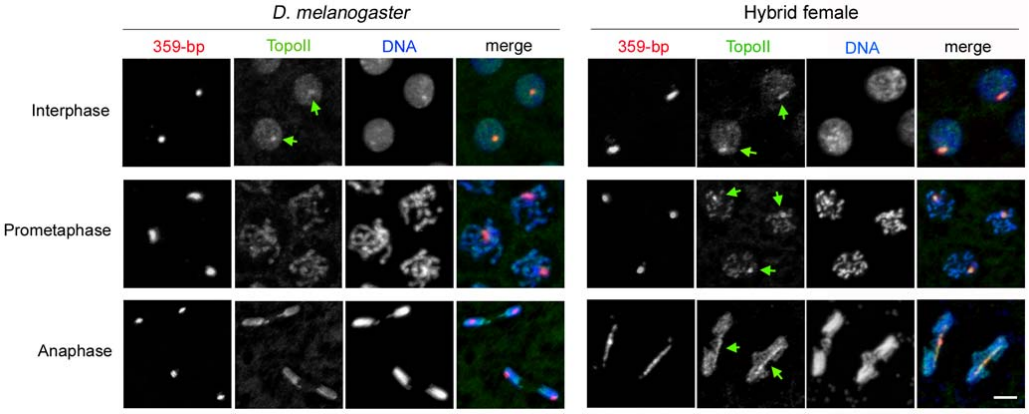
Topoisomerase II (TopoII) is mis-localized on the 359-bp satellite block during mitosis in hybrid female embryos. Green arrows indicate TopoII localized to the 359-bp satellite block. Scale bar is 5 µm.

We also analyzed the localization pattern of TopoII in hybrid female embryos. TopoII is the primary enzyme in *Drosophila* that decatenates newly replicated DNA strands and is also believed to be a structural component of condensed chromatin [41],[42]. In control *D. melanogaster* embryos, TopoII localized to 359-bp DNA during interphase and became more evenly distributed across the chromosomes through mitosis, with an occasional, slight enrichment on the 359-bp block at anaphase (Figures 5 and S6). However, in hybrid female embryos TopoII localized to the 359-bp satellite block during interphase but remained highly and consistently localized to this DNA through mitosis (Figures 5 and S6). We observed no TopoII foci during anaphase in hybrid male or *D. simulans* male or female embryos (Figure S7), in which the 359-bp satellite block is absent, further supporting the conclusion that abnormal TopoII persistence in hybrid female embryos occurs specifically on the 359-bp satellite block. This finding and the observed stretched and lagging 359-bp DNA together indicate the presence of a structural defect in this heterochromatin block that prevents chromatid separation.

## Discussion

### Mechanism of Heterochromatin-Induced Hybrid Lethality

We have shown that hybrid females produced from *D. simulans* mothers and *D. melanogaster* fathers die during early embryogenesis because of widespread mitotic defects induced by separation failure of the 359-bp satellite block on the paternal X chromatids.

Elegant genetic experiments by Sawamura and colleagues first suggested that hybrid female lethality is caused by a *D. melanogaster* heterochromatic locus *Zhr* [25],[36]. Genetic mapping localized *Zhr* to a pericentric region of the X chromosome containing the 359-bp satellite block. Because it is otherwise unprecedented for a heterochromatic locus to cause HI, this finding raised the key question of how *Zhr* kills wild-type female hybrids.

We suggest that our results strongly support the conclusion that the 359-bp satellite block directly and specifically causes hybrid lethality, as opposed to alternative possibilities outlined in the Introduction, including indirect effects on other genomic regions. First, we found that hybrid female embryos exhibit large chromatin bridges during anaphase and telophase of mitotic cycles 10–13 that are almost exclusively comprised of DNA from the 359-bp satellite block on the *D. melanogaster* X chromosome. While these bridges also included some flanking AATAT satellite, a large amount of this satellite is present on the *Zhr* ^1^ chromosome, which segregates normally, arguing against the AATAT satellite being causal for lethality. The small amount of lagging AATAT DNA detected in hybrid female embryos may result from over-catenation and tangling of AATAT DNA with the 359-bp DNA due to mis-localized TopoII (see below) when the chromatin is uncondensed, and is thus likely a secondary effect. Second, the entire multi-mega-bp satellite block appears to be stretched across the metaphase plate, suggesting that hybrids suffer from a structural defect in this block. Third, concomitant with these chromatin bridges we observed mitotic asynchrony and other aberrations that have been found in *D. melanogaster* mutants that have chromatin bridges [30]–[32]. In these cases, the lagging chromatin prevents complete separation of the daughter chromosome sets, thus inhibiting further mitotic divisions. Fourth, we found that all of these mitotic defects are suppressed in the *Zhr* ^1^ mutant, which lacks the 359-bp satellite block, and are induced on a Y chromosome that contains a translocation of the 359-bp satellite block and causes hybrid lethality in males, albeit with incomplete penetrance.

### What Is the Structural Basis of the Stretched and Lagging 359-Bp Satellite Block?

An important clue comes from our finding that TopoII localizes abnormally to the 359-bp satellite block during mitosis in hybrid female embryos. Both the DNA-decatenating and structural roles of TopoII are believed to be essential for normal chromatid separation [42]. These observations suggest several possible explanations for the hybrid phenotype. One possibility is that X chromatid separation failure results directly from incompatibility between *D. simulans* TopoII and the *D. melanogaster* 359-bp satellite block. TopoII is well conserved in the *melanogaster* subgroup (*D. melanogaster* and *D. sechellia* TopoII proteins are 95.6% identical based on analysis of the full-length *D. melanogaster* TopoII and the ∼98% of TopoII sequence available for *D. sechellia*; only ∼78% of *D. simulans* TopoII sequence has been assembled), arguing that TopoII is not a primary incompatibility factor. Nevertheless, future transgenic experiments will be important for testing this idea.

Alternatively, the persistence of TopoII may reflect a response to incomplete replication of the 359-bp satellite block as a result of incompatibilities with the *D. simulans* replication machinery. Extensive and unresolved tangling of daughter DNA strands would prevent separation of the *D. melanogaster* X chromatids at anaphase. Our observations suggest that the centromeres of the X chromatids are active and pulled toward the spindle poles, thus creating tension that results in stretching of the 359-bp satellite block. However, it is unlikely that an incompatibility with the *D. simulans* replication machinery is the primary cause because the first nine mitotic divisions occur normally, suggesting that replication during these divisions is normal.

A third possibility is that abnormal TopoII persistence may result from improper heterochromatin formation of the 359-bp satellite block. Chromatid separation failure in hybrid females occurs during mitotic cycles 10–13 when heterochromatin initially forms. This process involves visible changes in chromatin condensation and localization of HP1 to pericentric and telomeric regions, and precedes the major transition from maternal to zygotic gene expression [43],[44]. Our data thus argue that chromatin bridges and lethality result from a failure of heterochromatin formation at the 359-bp satellite block. Defective heterochromatin formation may lead to other effects such as improper replication and tangling of daughter DNA strands, ultimately causing failure of chromatid separation.

### Does the 359-Bp Repeat Cause Hybrid Embryonic Lethality?

What DNA sequences are responsible for these *Zhr* lethal effects? Our data argue strongly against the possibility that *Zhr* corresponds to an unknown protein-coding gene embedded within the 359-bp satellite block. Such a hypothetical gene would have to have the highly unusual property of causing mis-segregation of the entire satellite block in which it happens to be located. Furthermore, there is unlikely to be sufficient time to transcribe such a gene to cause lethality since the mitotic defects occur during the early stages of embryogenesis when zygotic transcription is minimal [45].

Previous genetic studies by Sawamura and colleagues led them to propose that the *Zhr* hybrid lethal effect is caused by repetitive elements in the pericentric region of the *D. melanogaster* X [27],[36],[46]. By assaying a series of X pericentric deletions and duplications of different sizes they further concluded that the lethality is quantitative, and correlates with the amount of pericentric heterochromatin present. Several *Zhr^−^* stocks contained less 359-bp repeat DNA than a wild-type *Zhr^+^* stock [46], a finding consistent with the possibility that a dosage threshold of the 359-bp repeat causes hybrid lethality. However, they excluded the 359-bp repeat (referred to as the 1.688 g/cm^3^ satellite) as causing hybrid lethality because two copies of two different mini-chromosomes containing 359-bp repeats did not induce hybrid lethality [46]. The authors inferred that the double dosage of these mini-chromosomes would contain more 359-bp repeats than a single dose of another mini-chromosome that did reduce viability, thus concluding that dosage of the 359-bp repeat does not correlate with hybrid lethality. We suggest two caveats to this conclusion. First, while Southern blots suggested that differences in the abundance of 359-bp repeats are present in the mini-chromosome stocks, quantitative methods were not used to estimate the abundance of 359-bp repeats that are present specifically on the mini-chromosomes. Second, increased dosage of 359-bp repeats may induce lethality only when present as a single block on a single chromosome, and not when dispersed over multiple chromosomes. Subsequent experiments, however, showed that a different mini-chromosome can induce lethality when in two doses [27]. Although the cause of the discrepancy between the two studies remains unclear, they were later interpreted to indicate that either the 359-bp repeat or other repetitive elements are causing hybrid lethality [47].

Our experiments do not allow us to rule out the possibility that other repetitive elements present in the 359-bp satellite block and also unique to the *D. melanogaster* X chromosome contribute to hybrid lethality. Various TEs are known to be interspersed within the 359-bp satellite block [48]–[50], however none are specific to the X chromosome and thus cannot account for the X chromosome-specific segregation defects we observed. In contrast, several lines of evidence argue that the 359-bp repeat is the primary contributor to the *Zhr* hybrid lethal effect. First, the 359-bp repeat is among the most highly abundant satellite repeats in the *D. melanogaster* genome [15]. And while there are scattered repeats along the *D. melanogaster* X chromosome [51], the vast majority are found in the proximal pericentric heterochromatin where *Zhr* maps. Second, the 359-bp satellite is essentially species-specific, being ∼50-fold more abundant in *D. melanogaster* than in *D. simulans* and highly diverged in primary sequence of its monomers between these species [15],[34]. This species-specificity makes it an attractive candidate in evolutionary models that can account for the nonreciprocal nature of the F1 female lethality in *D. melanogaster*/*D. simulans* hybrids (see below). Third, the entire 359-bp satellite block becomes stretched during mitosis in hybrids. If another unidentified repetitive element is causing this effect, it must be distributed evenly across the entire 359-bp satellite block and not on other chromosomes.

Our experiments are consistent with the idea that large amounts of the 359-bp repeat present in one block are required to induce chromosome segregation defects. First, the related 353-bp, 356-bp, and 361-bp repeats, located in much smaller amounts on *D. melanogaster* Chromosome 3, do not induce any observable mis-segregation in hybrids. This observation could mean that only the 359-bp monomer is capable of disrupting chromosome segregation, or, alternatively, that large amounts of this satellite class are required to cause lethality. Second, translocation of approximately half of the X-linked 359-bp satellite block to the Y chromosome resulted in lagging Y chromatin and hybrid male lethality that are proportionally less penetrant than the effects induced by the full-length 359-bp satellite block in hybrid females.

The multi-mega-bp size of the 359-bp satellite block precludes definitive genetic tests using transgenic methods. We suggest, however, that the available evidence strongly supports the hypothesis that the 359-bp repeat is the sequence element within the 359-bp satellite block that is the cause of the *Zhr* hybrid lethal effect.

### Insights into the Maternal Side of Hybrid Female Lethality

The fact that hybrid females are lethal when produced from *D. simulans* mothers and *D. melanogaster* fathers but viable when produced from the reciprocal cross clearly demonstrates the involvement of a maternal effect in this incompatibility. Our results can explain this maternal effect as follows. First, we suggest that the 359-bp satellite block requires maternal factor(s) in order to be packaged as heterochromatin during normal embryonic development in *D. melanogaster*. Second, *D. simulans* does not require such factors because it does not contain the 359-bp satellite block. These factors are therefore diverged in or absent from *D. simulans*. Third, in F1 hybrids from *D. simulans* mothers, the paternally inherited *D. melanogaster* 359-bp block fails to be packaged properly as heterochromatin because the requisite maternal factors are missing or functionally diverged.

Our proposal that the heterochromatin structure of the 359-bp satellite block is defective in hybrid females provides several promising hypotheses to explain the molecular nature of this incompatibility and the underlying maternal component(s). Satellites and other repetitive DNA elements are normally packaged into heterochromatin with general heterochromatin factors such as HP1 [52],[53], and, in some cases, with repeat-class-specific proteins like D1 [40], GAGA [54], and Prod [55]. These findings suggest a model in which high divergence in both the primary sequence and the abundance of repeat elements leads to incompatibilities with DNA-binding proteins expressed in the hetero-specific maternal cytoplasm. We tested D1 as a candidate maternal incompatibility factor because of its specific association in larval tissues with AT-rich satellite DNA, including the 359-bp repeat, but found that D1 does not localize to the 359-bp satellite block during early embryogenesis. Additional studies will be required to identify new candidate proteins that associate with the 359-bp satellite block in embryos in order to further test this model.

Alternatively, hybrid female lethality may be due to a mechanism involving small RNAs. In the yeast *Schizosaccharomyces pombe* and other organisms, RNA interference pathways and small RNAs are required for heterochromatin formation [56]. Recent studies have identified 359-bp satellite-derived small RNAs in the maternal cytoplasm of *D. melanogaster* [57],[58], raising the possibility that they may be required for initial heterochromatin formation and epigenetic silencing of the 359-bp satellite block during early embryogenesis. We have proposed that hybrid female lethality occurs owing to the absence of 359-bp–derived small RNAs in the *D. simulans* maternal cytoplasm [59]. According to this model, hybrid females from the reciprocal cross are viable because these small RNAs are present in the *D. melanogaster* maternal cytoplasm.

Regardless of the mechanistic basis of the maternal effect, it remains interesting that only the 359-bp satellite block is aberrant in hybrids, even though other satellite DNAs show significant differences in abundance and location between *D. melanogaster* and *D. simulans* [15]. Larger, more complex satellite repeats such as the 359-bp repeat may be more prone to cause HI than simple-repeat satellites, which are also known to vary in abundance between *Drosophila* species [15], because of their greater repeat sequence variation and perhaps, more complex heterochromatic structure.

### Species-Specific Heterochromatin as a Basis for Exceptions to Haldane's Rule

The incompatibility between the *D. melanogaster* X-linked 359-bp satellite block and the *D. simulans* maternal cytoplasm likely explains why the lethality from this cross violates Haldane's rule [24]. In this cross only hybrid females are lethal because they inherit the paternal (*D. melanogaster*) X chromosome carrying the 359-bp block, while viable hybrid males inherit the paternal Y. Although Haldane's rule is observed in many taxa, it is frequently violated in other *Drosophila* hybridizations that produce unisexual lethality, and in several of these cases, hybrids die during embryogenesis from a paternal X-linked locus [60]. We propose that paternally inherited X-linked heterochromatic repeats are strong candidates for causing hybrid female lethality in these interspecific crosses.

### Repeat-Sequence Evolution and Its Consequences

Much of repetitive DNA evolution is likely governed by neutral evolutionary processes [61]. However, variation in satellite DNAs can also be driven by their ability to mediate genetic conflicts such as segregation distortion [62]. In the *D. melanogaster* segregation distorter (SD) system, sperm bearing high-copy alleles of the 240-bp Responder (Rsp) satellite are targeted for destruction while sperm with low-copy alleles are immune to this effect [63], thus selecting strongly against high-copy alleles. Variation in satellite DNA abundance may also be influenced by meiotic drive in female meiosis [64]–[66]. Female meiosis is particularly prone to meiotic drive because only one of the four meiotic products becomes the maternal pronucleus of the egg. This situation creates an opportunity for competition among chromatids to gain access to the egg, with variation in centromeres being a prime candidate for mediating such antagonism among chromosomes. A recent example of this phenomenon was found in Mimulus, in which distinct centric or pericentric repeat alleles appear to confer a substantial chromosomal transmission advantage during female meiosis in conspecific crosses and a more extreme advantage in interspecific hybrids [67]. Meiotic drive and other types of genetic conflict may therefore be important for causing rapid evolution of repetitive sequences within species and fixed differences between closely related species. Our data demonstrate that as these interspecific differences accumulate, repetitive sequences can inhibit chromosome segregation in hybrids and thus directly cause reproductive isolation.

## Materials and Methods

### *Drosophila* Lines and Interspecific Crosses

Strains used were: wild-type *D. simulans* C167.4 and NC48S [68], and *white* ^501^ (SA32) (made by introgressing the *white* ^501^ allele into an isofemale South African *D. simulans* strain that mates well with *D. melanogaster*, provided by C. Aquadro), and wild-type *D. melanogaster* Oregon R and Canton S. The hybrid rescuing *Zhr* ^1^ chromosome (full genotype is *XYS.YL.Df(1)Zhr)* is described in [25] and the *Zhr* ^+^ Y chromosome (full genotype of strain is *Ts(1Lt;Ylt)Zhr/Dp(1;Y)y^+^*) is described in [36]; the structures of both chromosomes are shown in Figure 2A. Crosses were conducted by combining 40–50 0–8-h-old virgin *D. simulans* females and 60–80 12–24-h-old *D. melanogaster* males. Flies were allowed to mate for 48 h in a 25°C incubator with a 12-h light/12-h dark cycle prior to embryo collection.

### Embryo Collection and Fixation

Embryos were collected on grape juice agar plates [69] over a 3-h period and dechorionated in 50% bleach. Immediately afterward, embryos were fixed for 10 min in 4% EM-grade paraformaldehyde (Electron Microscopy Sciences) and heptane (Sigma-Aldrich) and then devitellinized in 100% methanol (Sigma-Aldrich). Fixed embryos were hydrated by using a series of methanol∶1×PBTA buffer solutions (9∶1, 5∶5, 1∶9 by volume) and treated with RNaseA (Sigma-Aldrich) at 37°C for 2 h before carrying out FISH or immuno-staining.

### FISH and Immuno-Staining

The following sequences were used for FISH probes: (TTT-TCC-AAA-TTT-CGG-TCA-TCA-AAT-AAT-CAT) recognizing the 359-bp satellite block on the *D. melanogaster* X as well as minor variants on *D. melanogaster* Chromosome 3 [34]; (AAT-AC)_6_ recognizing a small block of this sequence on the *D. melanogaster* Y [70]; (AAT-AT)_6_ recognizing large amounts of this sequence on *D. melanogaster* and *D. simulans* Chromosome 4 as well as a small region on the *D. melanogaster* X [15],[70]; (AAG-AG)_6_ recognizing primarily a large block of this sequence on the *D. melanogaster* 2 and a small block on the *D. simulans* X [15]; (AAT-AAC-ATA-G)_3_ recognizing a single block of this sequence on *D. melanogaster* Chromosomes 2 and 3 [70]; and (AAT-AAA-C)_4_ recognizing a single region on the *D. simulans* Y (S. Maheshwari, personal communication). These sequences were chemically synthesized (MWG Biotech) and modified at the 5′ terminus with either fluorescein, Cy3, or Cy5 for fluorescent detection. The euchromatic FISH probe was made by random priming of BAC DNA (BACR19J01 from CHORI BACPAC Resources) using the BioPrime DNA Labeling System (Invitrogen), which incorporates amino-allyl-dUTP into amplified DNA products (protocol from A. Minoda). These products were sonicated into 100–150-bp fragments, cleaned with MiniElute columns (Qiagen), and conjugated with an Alexa546 fluorophore reactant group (Invitrogen). The labeled probe was ethanol-precipitated twice before hybridization. The *D. simulans* 360-bp probe was made by end-labeling a 360-bp PCR product with poly-amino-allyl-dUTP and Terminal Transferase (Roche). This product was amplified from *D. simulans* C167.4 genomic DNA using the following primers that recognize the *D. simulans* 360-family monomer repeat [34]: forward ACT-CCT-TCT-TGC-TCT-CTG-ACC-A and reverse CAT-TTT-GTA-CTC-CTT-ACA-ACC-AAT-ACT-A. The rDNA probe was made by using a 1,200-bp fragment of five tandem repeats of the rDNA IGS region cloned into pBluescript (construct was a gift from G. Bosco). This fragment was digested out of the plasmid using the restriction enzymes KpnI and SacI, digested into ∼150-bp fragments with AluI and MseI, and purified using the Qiaex II gel extraction kit (Qiagen). End-labeling was conducted as described above.

FISH was performed as described [70] with minor modifications. Overnight incubation of fixed tissues with probe was conducted in a thermocycler with a denaturation temperature of 92°C for 3 min and a hybridization temperature of 32°C overnight. This lower hybridization temperature (standard is 37°C) was important for detecting the 359-bp variant sequences on *D. melanogaster* Chromosome III and did not result in excessive nonspecific hybridization. Three additional 10-min washes in 50% formamide/2×SSCT were performed to maximize the removal of any nonspecifically bound probe. For immuno-FISH experiments, fixed embryos were hybridized with primary and secondary antibodies as described [69]. Embryos were subsequently fixed in 4% paraformaldehyde for 30 min, and washed 3× with PBTX and 3× with 2×SSCT. FISH was then conducted as described above. For immuno-cytological or immuno-FISH experiments, Rat anti-HA (Roche 3F10) and rabbit anti-D1 antibodies (a gift from E. Käs) [39] were used at 1∶100 and 1∶1,000, respectively. Rabbit anti-TopoII (a gift from T. Hsieh) [71] and mouse anti-PH3 (Santa Cruz Biotechnology) were used at 1∶1,000. Alexa555 anti-rabbit, Alexa555 anti-rat, and Alexa633 anti-mouse secondary antibodies were used at 1∶300 (Molecular Probes). Tissue preparation, FISH, and D1 immuno-staining of larval brain chromosomes were performed as described [39]. DNA was stained by using either OliGreen or TO-PRO-3 iodide (Molecular Probes) for embryos and Vectashield containing DAPI (Vector Laboratories) for brain tissues. All imaging was conducted at the Cornell Microscopy and Imaging Facility, using either a Leica DM IRB confocal microscope or an Olympus BX50 epifluorescent microscope. Confocal images were generated by using sequential collection of each wavelength to eliminate bleed-through of fluorophores and generated as maximum projections of multiple scans. Images were processed using Photoshop (Adobe, version 7.0). Contrast and brightness changes, when used, were applied globally across the image. Images shown in the figures were taken from either hybrid or pure species embryos produced from C.167.4 and/or Canton S strains, unless otherwise specified. However, cytological analyses were also performed on hybrid embryos produced from other parental lines shown in Table 1 for verification of the observed phenotypes.

### Western Blotting

0–3-h embryos from C167.4 and Canton S flies were collected as described above and washed in 1× PBS buffer. 50 µl embryos from each strain were lysed in an equal volume of 2× SDS Sample Buffer [72] and boiled for 5 min. Five µl of protein extracts were separated on a 10% polyacrylamide gel for 1 h at room temperature and 100 V. Proteins were transferred to a nitrocellulose membrane overnight at 4°C and 20 V, blocked with 5% powdered milk, and then blotted overnight at 4°C with anti-D1 serum (1/10,000 dilution). Membranes were then blotted with goat anti-rabbit HRP antibodies (1/5,000 dilution; Jackson) for 1 h at room temperature. HRP was detected using ECL Western blotting substrate (Pierce).

### *D1* Constructs and Transformation

The complete *D. melanogaster* and *D. simulans D1* coding sequences were PCR amplified from Canton S and C167.4 adult cDNA, respectively, by using the following primers: for *D. melanogaster*, forward CAC-CAT-GGA-GGA-AGT-TGC-GGT-AAA-GAA-G and reverse TTA-GGC-AGC-TAC-CGA-TTC-GG; for *D. simulans*, forward CAC-CAT-GGA-AGA-AGT-TGC-GGT-AAA-GAA-G and reverse TTA-GGC-AGC-TAC-CGA-TTC-GG. The resulting fragments were cloned into the pENTR/D-TOPO vector (Invitrogen). Positive clones were fully sequenced to confirm the absence of any errors. Each sequence was recombined into the pPHW plasmid downstream of the *UAS* transcriptional activation sequence and in frame with an N-terminal 3× HA peptide (Murphy collection; described at http://www.ciwemb.edu/labs/murphy/Gateway%20vectors.html). Additionally, the *attB* sequence was subcloned into this plasmid for site-specific integration into the *D. melanogaster* strain *y^1^ w^67c23^; P{CaryP}attP2* [73]. The resulting transformants were crossed with the strain *w; P{matα4-GAL-VP16}V37* (Bloomington Stock Center) for expression of the HA-tagged *D1* in the early embryo.

## Supporting Information

Figure S1 **Lagging and stretched 359-bp satellite DNA in hybrid female embryos.** Left panel, dividing chromosomes in a hybrid female embryo with lagging and stretched 359-bp DNA at the metaphase plate. Middle panel, dividing chromosomes in a hybrid female embryo with segregating but stretched 359-bp satellite blocks. Right panel, dividing chromosomes in a control *D. melanogaster* female embryo. Nuclei in all panels are in late anaphase. Scale bar is 5 µm.
(0.31 MB TIF)

Figure S2 **Mapping of FISH probes and D1 protein on larval brain metaphase chromosomes, and analysis of** ***Zhr*** **^1^ chromosome structure**. (A) *D. melanogaster*, hybridized with probes that recognize the satellite sequences AATAACATAG on Chromosomes 2 and 3 and AAGAG primarily on Chromosome 2. Yellow arrows indicate Chromosome 2 homologs and white arrows indicate Chromosome 3 homologs. (B) *D. simulans*, hybridized with same probes in (A). White arrows indicate X chromosomes. (C) *D. melanogaster*, hybridized with the 359-bp probe. White arrows indicate the 359-bp satellite block on the X chromosome and yellow arrows indicate small regions of related monomer repeats (353-bp, 356-bp, and 361-bp) on Chromosome 3. (D) *D simulans*, hybridized with the same probe in (C). White arrows indicate the X chromosomes. No hybridization is detected. (E) *D. melanogaster*, hybridized with the BACR19J01 probe. White arrows indicate the tips of the X euchromatic arms. (F) *D. melanogaster Zhr* ^1^, hybridized with the 359-bp probe. White arrows indicate the compound-XY chromosomes that are devoid of 359-bp DNA. Yellow arrows indicate the related monomer repeats on Chromosome III. Image was taken with a high gain setting to detect the related monomer repeats and to insure the absence of 359-bp DNA signal on the compound-XY chromosome. (G) *D. melanogaster*, hybridized with probes for the AATAT and AAGAG satellites. A small amount of each satellite is present around the centromere and in the small arm of the X chromosome (white arrow). (H) *D. melanogaster Zhr* ^1^, hybridized with the same probes in (G). Yellow and white arrows indicate the Y and compound-XY chromosomes, respectively. The AAGAG and AATAT satellites on the compound-XY chromosome are derived from the long arm of the Y chromosome (regions indicated by the arrows are identical). (I) *D. simulans*, hybridized with probes for the AATAT and *D. simulans* 360-family satellites (S360). Yellow arrows indicate Chromosome 4 homologs, which contain little AATAT sequence, and white arrows indicate the X chromosomes, which contain little 360-family sequence and no AATAT sequence. There are much smaller amounts of these satellites across the genome in *D. simulans*, compared to *D. melanogaster*. (J) *D. melanogaster*, immuno-stained with anti-D1 antibodies. DNA is blue in all panels.
(1.26 MB TIF)

Figure S3 **Normal segregation of minor satellite variants 353-bp, 356-bp, and 361-bp in hybrid female embryos.** White arrows indicate the small region of the satellite variants on the left arm of *D. melanogaster* Chromosome 3. Scale bar is 3 µm.
(0.29 MB TIF)

Figure S4 **Partial mis-segregation of the** ***D. melanogaster*** **X-linked AATAT DNA in a hybrid female embryo.** This DNA segregates toward the spindle poles during anaphase (white arrows in panel (B)). X-linked AATAT sequence is also present in the lagging chromatin at the metaphase plate (yellow arrowhead). DNA is blue in panel (C). Scale bar is 3 µm.
(0.46 MB TIF)

Figure S5 **Western blot of D1 in** ***D. simulans*** **(lane 1) and** ***D. melanogaster*** **(lane 2) embryo extracts.** In both species, anti-D1 recognizes a band of approximately 60 kDa (black arrow).
(0.18 MB TIF)

Figure S6 **TopoII localizes aberrantly during mitosis in hybrid female embryos.** Wide fields show multiple nuclei in control *D. melanogaster* and hybrid female embryos. In *D. melanogaster*, TopoII localizes broadly across the chromosomes during metaphase. In hybrid females, TopoII is more enriched at a single locus during metaphase (white arrows); this region is the 359-bp satellite block as identified in our immuno-FISH experiments (see Figure 5). This localization difference is also present but not as prominent during anaphase (white arrows). Scale bar is 10 µm.
(2.14 MB TIF)

Figure S7 **TopoII is distributed evenly across the chromosomes during anaphase in the absence of the 359-bp satellite block.** Top row, anaphase spindles in a hybrid male embryo. In this experiment, sex was identified by normal chromosome segregation, which does not occur in female hybrid embryos. Bottom rows, anaphase spindles in *D. simulans* male and female embryos. A Y-specific FISH probe (AATAAAC) was used to distinguish embryo sex since the chromosomes segregate normally in both sexes of the pure species. Scale bars are 5 µm.
(0.56 MB TIF)

## Abbreviations

FISH: fluorescent in situ hybridization
HI: hybrid incompatibility
rDNA: ribosomal DNA
TE: transposable element
TopoII: Topoisomerase II
