# Supplementary figures and images for: Species-Specific Heterochromatin Prevents Mitotic Chromosome Segregation to Cause Hybrid Lethality in Drosophila
Source: PLoS Biol. 2009 Oct 27;7(10):e1000234. doi: 10.1371/journal.pbio.1000234 (PMC2760206; doi:10.1371/journal.pbio.1000234)

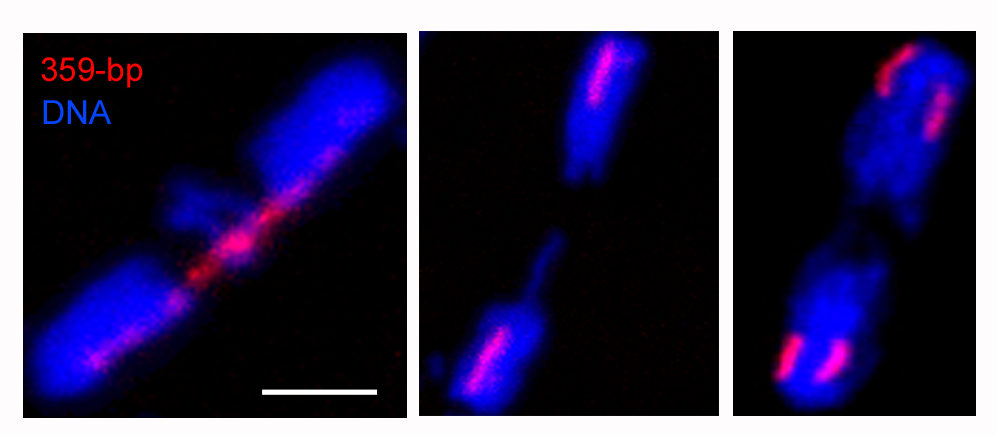

Supplement: Figure S1 — Lagging and stretched 359-bp satellite DNA in hybrid female embryos. Left panel, dividing chromosomes in a hybrid female embryo with lagging and stretched 359-bp DNA at the metaphase plate. Middle panel, dividing chromosomes in a hybrid female embryo with segregating but stretched 359-bp satellite blocks. Right panel, dividing chromosomes in a control D. melanogaster female embryo. Nuclei in all panels are in late anaphase. Scale bar is 5 µm. (0.31 MB TIF) [file pbio.1000234.s001.tif]

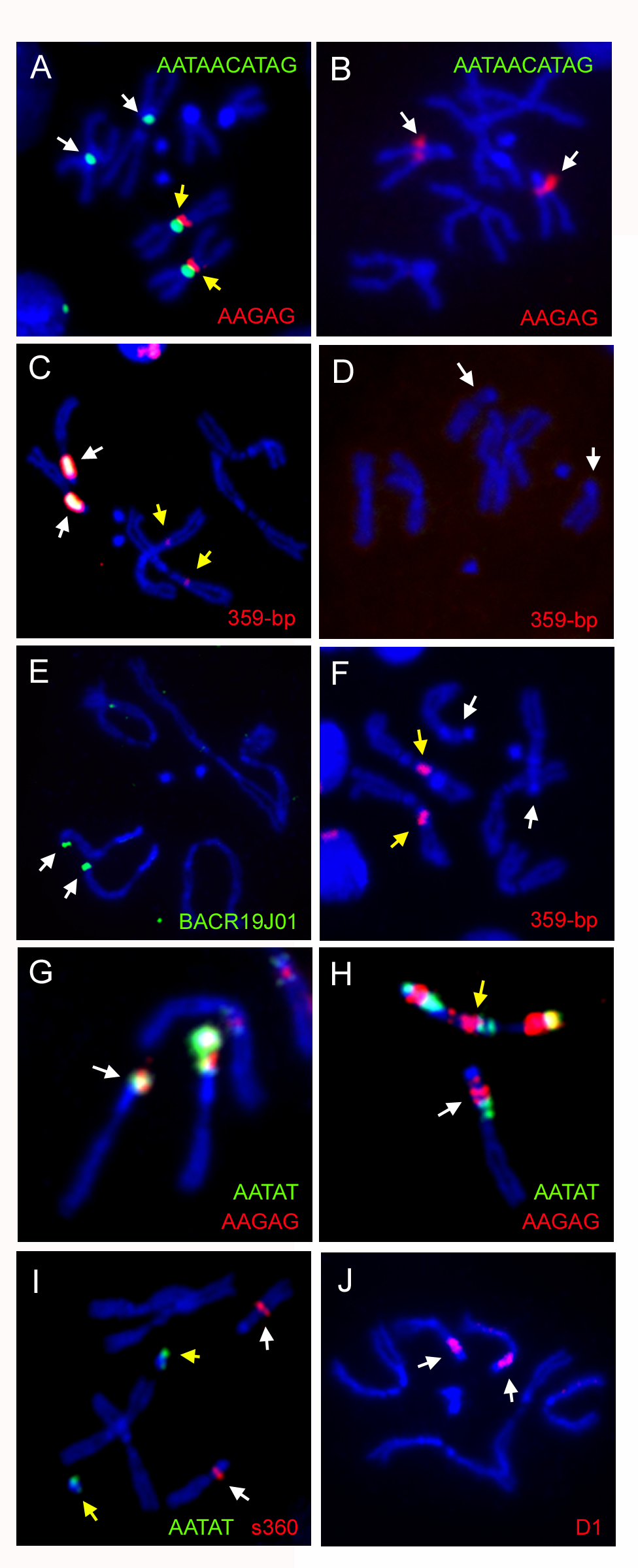

Supplement: Figure S2 — Mapping of FISH probes and D1 protein on larval brain metaphase chromosomes, and analysis of Zhr 1 chromosome structure. (A) D. melanogaster, hybridized with probes that recognize the satellite sequences AATAACATAG on Chromosomes 2 and 3 and AAGAG primarily on Chromosome 2. Yellow arrows indicate Chromosome 2 homologs and white arrows indicate Chromosome 3 homologs. (B) D. simulans, hybridized with same probes in (A). White arrows indicate X chromosomes. (C) D. melanogaster, hybridized with the 359-bp probe. White arrows indicate the 359-bp satellite block on the X chromosome and yellow arrows indicate small regions of related monomer repeats (353-bp, 356-bp, and 361-bp) on Chromosome 3. (D) D simulans, hybridized with the same probe in (C). White arrows indicate the X chromosomes. No hybridization is detected. (E) D. melanogaster, hybridized with the BACR19J01 probe. White arrows indicate the tips of the X euchromatic arms. (F) D. melanogaster Zhr 1, hybridized with the 359-bp probe. White arrows indicate the compound-XY chromosomes that are devoid of 359-bp DNA. Yellow arrows indicate the related monomer repeats on Chromosome III. Image was taken with a high gain setting to detect the related monomer repeats and to insure the absence of 359-bp DNA signal on the compound-XY chromosome. (G) D. melanogaster, hybridized with probes for the AATAT and AAGAG satellites. A small amount of each satellite is present around the centromere and in the small arm of the X chromosome (white arrow). (H) D. melanogaster Zhr 1, hybridized with the same probes in (G). Yellow and white arrows indicate the Y and compound-XY chromosomes, respectively. The AAGAG and AATAT satellites on the compound-XY chromosome are derived from the long arm of the Y chromosome (regions indicated by the arrows are identical). (I) D. simulans, hybridized with probes for the AATAT and D. simulans 360-family satellites (S360). Yellow arrows indicate Chromosome 4 homologs, which contain little AATAT sequence [file pbio.1000234.s002.tif]

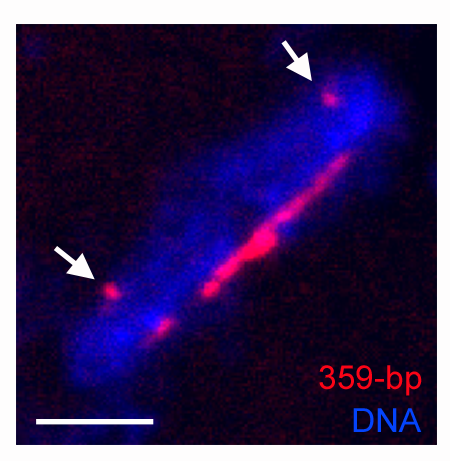

Supplement: Figure S3 — Normal segregation of minor satellite variants 353-bp, 356-bp, and 361-bp in hybrid female embryos. White arrows indicate the small region of the satellite variants on the left arm of D. melanogaster Chromosome 3. Scale bar is 3 µm. (0.29 MB TIF) [file pbio.1000234.s003.tif]

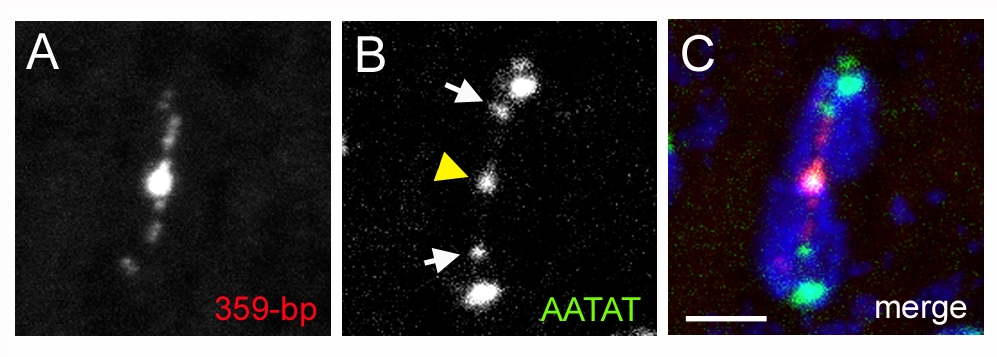

Supplement: Figure S4 — Partial mis-segregation of the D. melanogaster X-linked AATAT DNA in a hybrid female embryo. This DNA segregates toward the spindle poles during anaphase (white arrows in panel (B)). X-linked AATAT sequence is also present in the lagging chromatin at the metaphase plate (yellow arrowhead). DNA is blue in panel (C). Scale bar is 3 µm. (0.46 MB TIF) [file pbio.1000234.s004.tif]

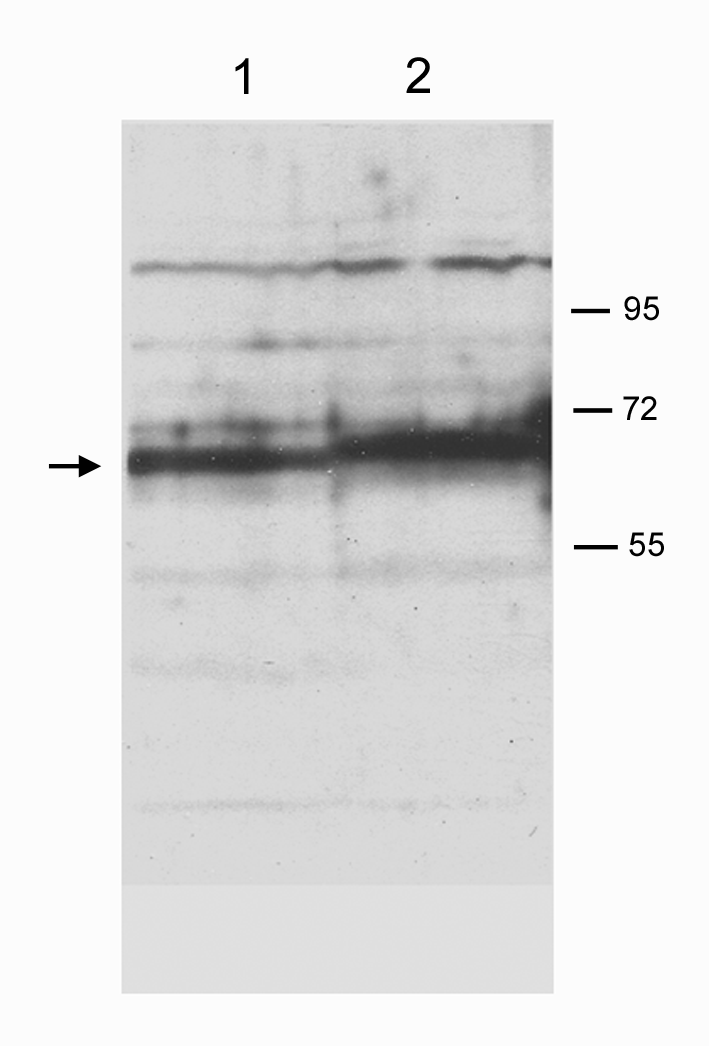

Supplement: Figure S5 — Western blot of D1 in D. simulans (lane 1) and D. melanogaster (lane 2) embryo extracts. In both species, anti-D1 recognizes a band of approximately 60 kDa (black arrow). (0.18 MB TIF) [file pbio.1000234.s005.tif]

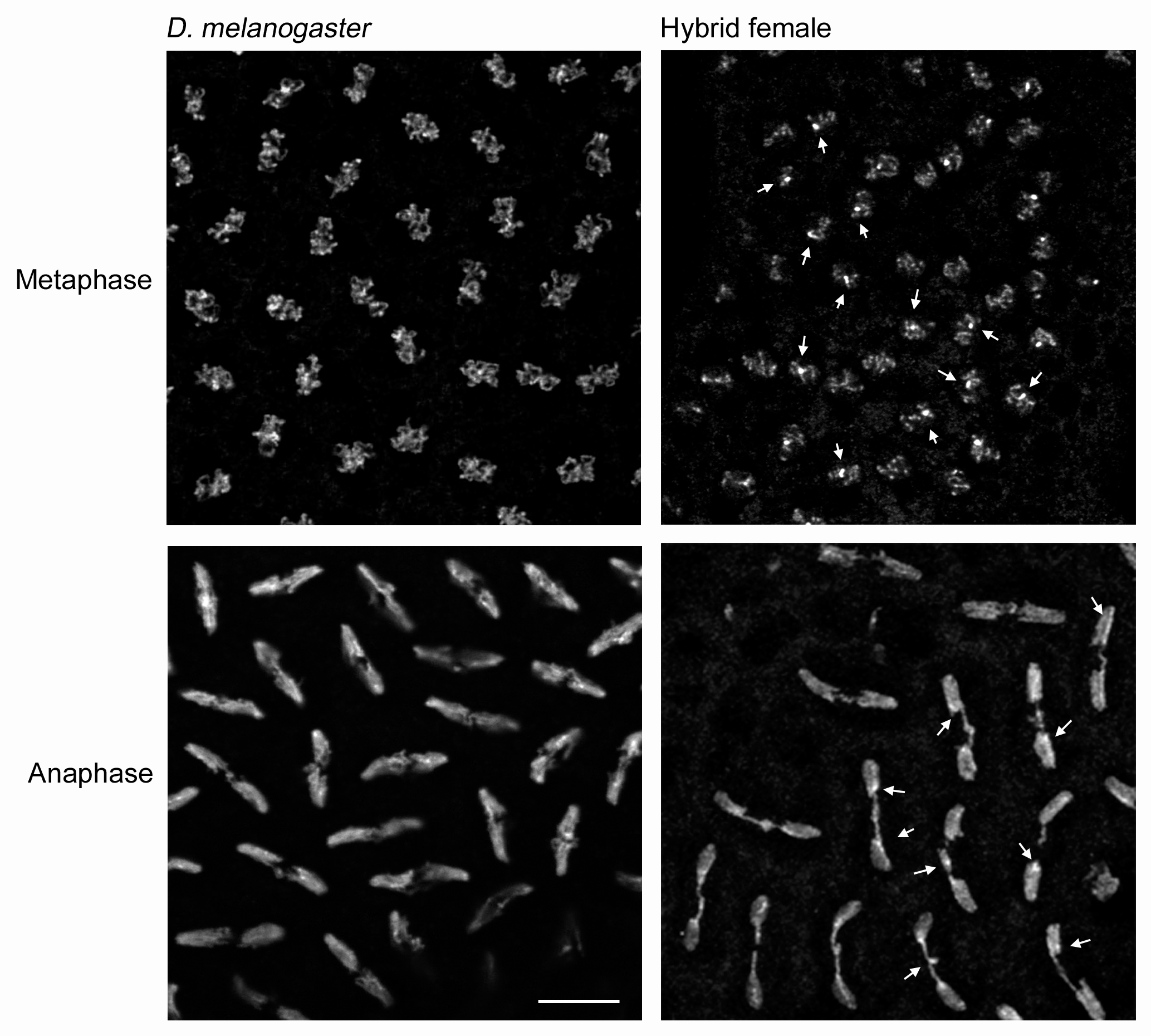

Supplement: Figure S6 — TopoII localizes aberrantly during mitosis in hybrid female embryos. Wide fields show multiple nuclei in control D. melanogaster and hybrid female embryos. In D. melanogaster, TopoII localizes broadly across the chromosomes during metaphase. In hybrid females, TopoII is more enriched at a single locus during metaphase (white arrows); this region is the 359-bp satellite block as identified in our immuno-FISH experiments (see Figure 5). This localization difference is also present but not as prominent during anaphase (white arrows). Scale bar is 10 µm. (2.14 MB TIF) [file pbio.1000234.s006.tif]

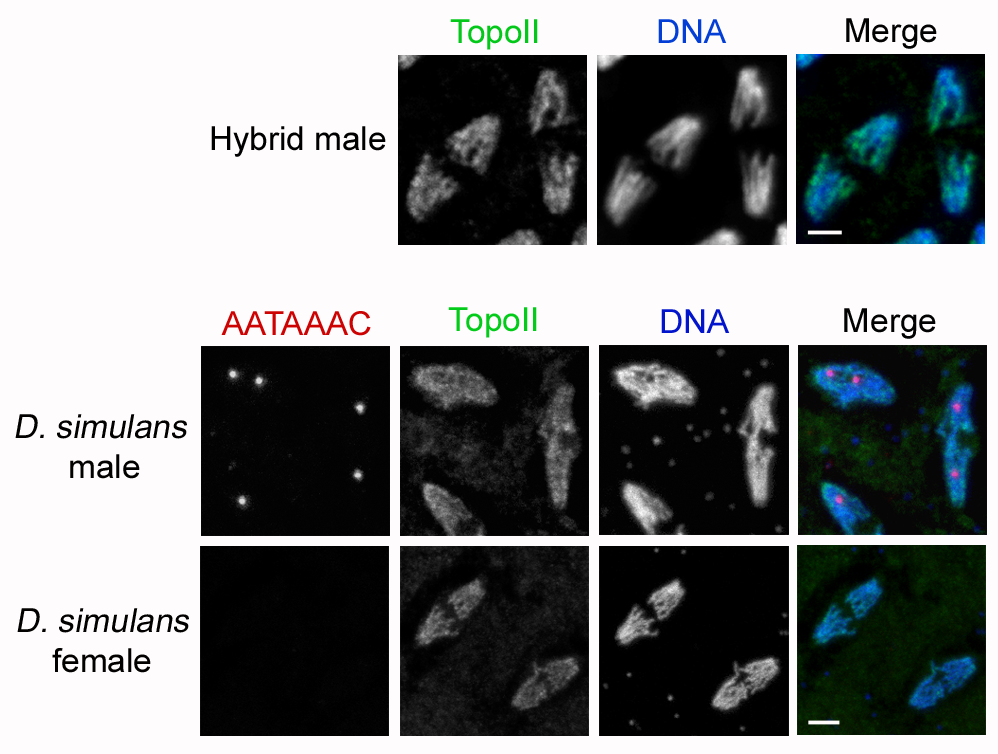

Supplement: Figure S7 — TopoII is distributed evenly across the chromosomes during anaphase in the absence of the 359-bp satellite block. Top row, anaphase spindles in a hybrid male embryo. In this experiment, sex was identified by normal chromosome segregation, which does not occur in female hybrid embryos. Bottom rows, anaphase spindles in D. simulans male and female embryos. A Y-specific FISH probe (AATAAAC) was used to distinguish embryo sex since the chromosomes segregate normally in both sexes of the pure species. Scale bars are 5 µm. (0.56 MB TIF) [file pbio.1000234.s007.tif]
